# Assessing the feasibility and acceptability of implementing emergency department quality standards in Palestine: a qualitative study

**DOI:** 10.1186/s12913-026-14199-6

**Published:** 2026-02-24

**Authors:** Abed Alr‘oof Bani Odeh, Lee Wallis, Motasem Hamdan, Willem Stassen

**Affiliations:** 1 University of Cape Town, Cape Town, ZA South Africa; 2 Al-Quds University, East Jerusalem, Occupied Palestinian Territory, Jerusalem, Palestine

**Keywords:** Emergency department, Quality standards, Emergency medicine, Implementation, Feasibility, Acceptability, Health system strengthening, Low- and middle-income countries, Palestine

## Abstract

**Background:**

Emergency departments are critical for timely, life-saving care, particularly in conflict-affected, resource-constrained settings such as Palestine. Despite national prioritization of healthcare quality, sustained workload pressures from routine emergencies and occupation-related injuries, combined with the absence of ED-specific quality standards, limit systematic improvement of emergency services.

**Methods:**

This qualitative study assessed the perceived feasibility and acceptability of context-specific EDQS in Palestine. Ten semi-structured interviews were conducted with ED physicians, nurses, and quality leaders from nine hospitals, and data were analyzed using thematic analysis to identify key themes and subthemes related to staff experiences and expectations regarding EDQS implementation.

**Results:**

Four main themes and 16 subthemes were identified, highlighting both the enabling factors and challenges related to the implementation of EDQS. Enablers included professional commitment, leadership support, and cultural alignment. In contrast, key challenges included resource constraints, training gaps, and political and financial instability. Participants perceived the EDQS as acceptable and potentially feasible under supportive implementation conditions.

**Conclusion:**

This study found that frontline ED staff perceive the EDQS as both feasible and acceptable within the Palestinian context, despite persistent challenges related to knowledge gaps, resource constraints, and resistance to change. Importantly, these perceptions reflect professional commitment rather than ideal implementation conditions. The successful roll-out of EDQS will therefore depend on targeted capacity building, optimization of available resources, and a phased implementation approach supported by leadership and continuous monitoring, rather than solely on the standards themselves.

**Supplementary Information:**

The online version contains supplementary material available at 10.1186/s12913-026-14199-6.

## Background

 Emergency departments (EDs) are critical entry points for timely, life-saving care, particularly in low-resource and conflict-affected settings. In Palestine, EDs operate under sustained pressure due to high patient volumes from routine emergencies and injuries related to ongoing Israeli occupation, combined with chronic resource constraints and political and financial instability [1]. These conditions contribute to overcrowding, prolonged waiting times, workforce strain, and variability in the quality of emergency care delivery [2, 3]. Evidence from Palestine further demonstrates the profound psychological and systemic impact of political violence on the population and health services, resulting in increased trauma burden and reduced system capacity [4].

Variability in emergency care quality is often exacerbated by the absence or inconsistent application of standardized quality measures. Differences in infrastructure, staffing levels, training, leadership, and adherence to best practices can lead to disparities in patient outcomes and experiences [5]. In response, there has been increasing emphasis globally on developing standardized emergency department quality standards (EDQS) to support equitable, safe, and efficient care delivery, particularly in resource-limited settings [6].

To address these challenges, a comprehensive set of EDQS tailored to the Palestinian context was recently developed and validated through an extensive literature review and expert consensus. The standards comprise 100 items across clinical and administrative domains, reflecting key aspects of emergency care delivery, patient safety, leadership, workforce, and resource management. While these standards have been rigorously developed and validated, their successful adoption depends not only on technical soundness but also on their feasibility and acceptability in real-world ED settings [5].

In implementation science, *feasibility* refers to the extent to which an intervention can be practically implemented within existing workflows, resources, and organizational structures, while *acceptability* reflects stakeholders’ perceptions of the intervention’s appropriateness and value. Frontline ED staff physicians, nurses, and quality leaders play a pivotal role in translating standards into practice, and their perspectives are central to understanding the conditions that enable or hinder implementation and sustainability. Examining their experiences is therefore essential before broader implementation or scale-up of EDQS [7, 8].

This study explored frontline emergency department staff perceptions of the feasibility and acceptability of implementing Palestine’s Emergency Department Quality Standards and identified perceived enablers, challenges, and recommendations for implementation.

Specifically, the study addressed the following questions:

1. How do frontline ED staff perceive the feasibility and acceptability of the EDQS?
2. What enablers and barriers influence EDQS implementation in Palestinian EDs?
3. What recommendations do staff propose to support effective and sustainable implementation?

## Methods

### Study design

This study employed a qualitative design to assess the feasibility and acceptability of implementing context-specific EDQS in Palestine among experienced ED staff and to understand their perspectives on the quality of healthcare services. The study gathered in-depth feedback through semi-structured interviews with open-ended questions, allowing participants to share their experiences and insights. Data were analyzed using thematic analysis to identify key themes and subthemes reflecting participants’ views on the EDQS implementation [7–9]. EDQS included in this study had been developed and validated in previous studies, totalling 100 standards [5].

**Fig. 1:**
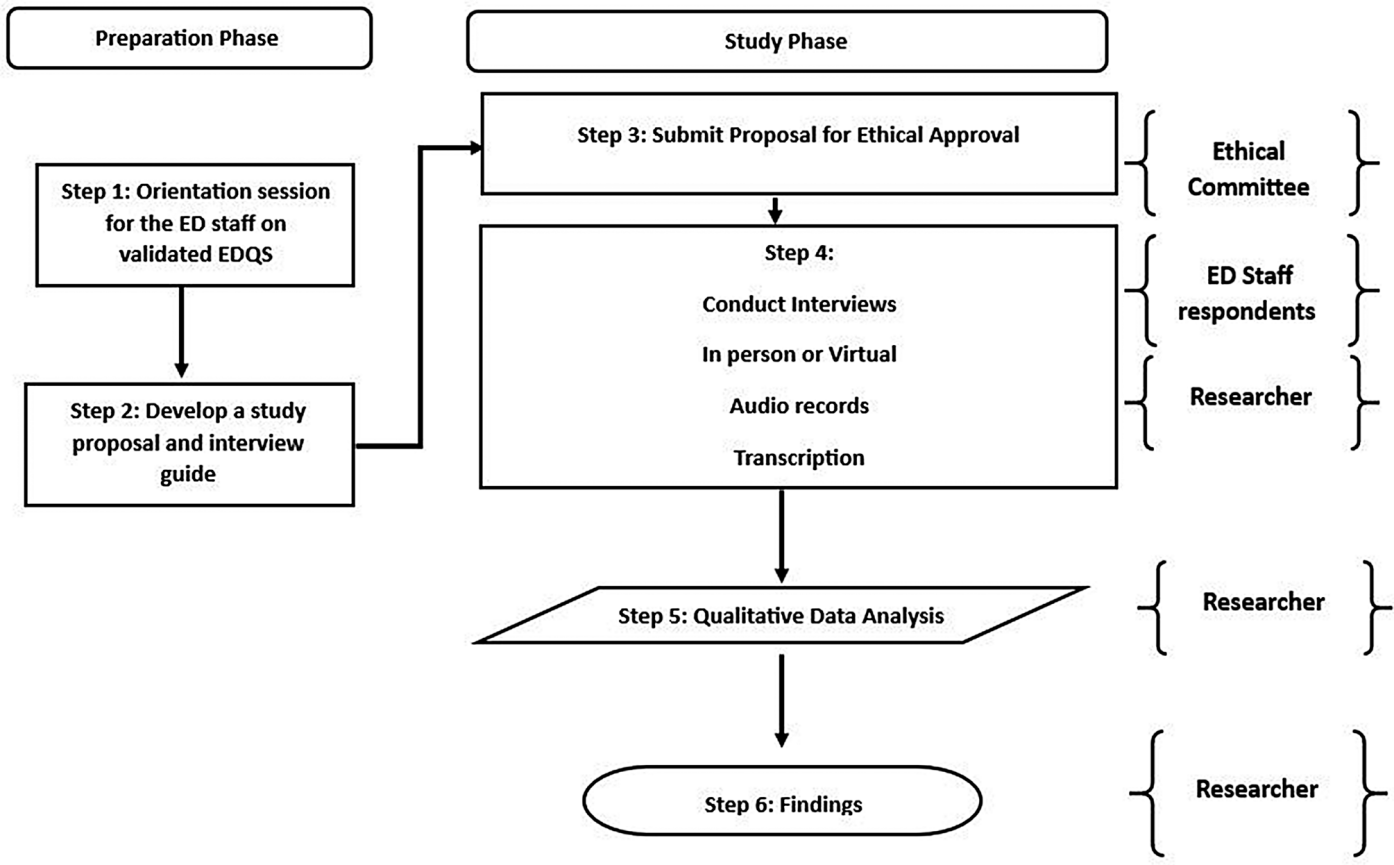
Research execution process

This study adhered to the Consolidated Criteria for Reporting Qualitative Research (COREQ) to ensure rigorous and transparent reporting of the research process and findings [10], Fig. 1.

Ethical approval from the Human Research Ethics Committee (HREC) at the University of Cape Town (HREC reference number 318/2024), and permission from the Palestinian Ministry of Health (PMoH) to conduct the study were obtained (MoH reference number (01/01-407 / DHM240407). Patients and the public were not involved in the design, conduct, reporting, or dissemination plans of this research.

### Setting and study population

The study was conducted in the EDs of eight public and two private hospitals across the northern, central, and southern regions of the West Bank, Palestine. These hospitals were selected to reflect variation in ownership (public and private), size, and service volume, and all provide 24-hour emergency care [11, 12].

### Inclusion and exclusion criteria

***Participants were eligible for inclusion if they***:

- Were currently working in an emergency department in one of the selected hospitals;
- Had a minimum of two years of experience in emergency care;
- Held a professional role relevant to emergency department operations, including physicians, nurses, or healthcare quality leaders/managers;
- Were able to communicate fluently in Arabic; and.
- Had sufficient familiarity with emergency department processes to provide informed perspectives on quality standards, even if they had not received formal training on the EDQS.

***Staff were excluded if they***:

- Had less than the minimum required experience in emergency care;
- Were not directly involved in ED clinical or quality-related activities; or.
- Were unavailable or unwilling to participate.

### Sampling strategy

A purposive sampling approach was used to deliberately capture diverse perspectives across professional roles (physicians, nurses, and quality leaders), levels of seniority, and facility types (public and private hospitals). This strategy was intended to ensure variation in experiences and viewpoints related to the feasibility, acceptability, and implementation of EDQS. Recruitment continued until thematic saturation was reached, which is defined as the point where no new codes, categories, or subthemes appeared in successive interviews during ongoing analysis [10, 11].

### Participants characteristics

The study included 10 participants from diverse leadership, clinical, and quality roles within EDs. The sample, consisting of nine males and one female, comprised four ED heads, one resident doctor, one ED head nurse, one ED nurse, and three Directors of Quality and Patient Safety (QPS).

Participants possessed significant professional experience, ranging from 5 to 20 years in emergency care and related fields, with varied backgrounds in medicine, nursing, and medical imaging. Their qualifications ranged from bachelor’s to master’s degrees, and several physicians were trained as EM specialists. The experience was distributed as follows: three participants had 17–20 years of experience, four had 12–17 years, and three had 5–8 years. Including participants from 8 public and 2 private EDs ensured diverse organizational contexts, offering comprehensive perspectives on the clinical, managerial, and quality governance aspects of implementing EDQS in Palestinian EDs. A profile of the study participants is given in Table 1.

**Table 1:** Participants (Interviewees) profile

Participant No.	Roles and Responsibilities	Experience Years	Background	Qualification	ED Type	Gender
P1	Resident Doctor	5	MD	Bachelor	Public	M
P2	Head of ED	18	MD	EM Specialist	Public	M
P3	Head of ED	7	MD	EM Specialist	Private	M
P4	QPS Director	17	Nurse	Master	Public	M
P5	ED Head Nurse	12	Nurse	Bachelor	Public	M
P6	QPS Director	12	Medical Imaging	Master	Public	M
P7	QPS Director	20	Nurse	Master	Public	F
P8	ED Nurse	20	Nurse	Master	Public	M
P9	Head of ED	17	MD	EM Specialist	Private	M
P10	Head of ED	8	MD	Bachelor	Public	M

ED = Emergency Department; QPS = Quality and Patient Safety; MD = Doctor of Medicine; EM specialist = Emergency Medicine specialist; PMoH = Palestinian Ministry of Health

### Recruitment and data collection

#### Researcher characteristics and reflexivity

The first author, a healthcare quality expert and the lead developer of the EDQS in this study, faced potential confirmation and social desirability biases regarding participants’ views on the standards.

To mitigate these potential risks, participation was voluntary, and confidentiality was emphasized. Participants were encouraged to openly discuss challenges and concerns about EDQS implementation using a semi-structured interview guide.

Data analysis involved iterative discussion with supervisory team members who were not involved in EDQS development, supporting reflexive interpretation and reducing single-researcher bias. Throughout the analytic process, reflexive memo-writing was used to critically examine how the researcher’s positionality may have influenced data generation and interpretation.

#### Research process

The research process began with orientation sessions for participants and ED leadership, followed by informed consent (Additional file 1). Semi-structured interviews were conducted with ED staff using open-ended questions, either in person (*n* = 1) or virtually (*n* = 9). Probing techniques were used to explore perceptions of EDQS feasibility and acceptability; no notable differences in data depth were observed between interview modes [13].

#### Recruitment and enrolment

Participants were invited to take part in the study voluntarily through official communication with their respective hospital management. Contact was established with the directors of the selected hospitals to coordinate staff participation and to facilitate the conduct of the interviews [10].

### Data collection

Data were collected using a semi-structured interview guide (Additional file 2); in-depth interviews were conducted with different key respondents from the governmental and private EDs [10, 12]. Participation was voluntary, and informed consent was obtained either through a written consent form or an audio-recorded oral consent.

Interviews were conducted and audio-recorded in Arabic, then transcribed verbatim. The first author, fluent in both Arabic and English, transcribed and translated the interviews into English. Accuracy was ensured by reviewing translated transcripts against the original Arabic recordings and cross-checking selected excerpts during analysis.

The estimated average duration of the interviews was approximately 35 min. An interview guide was developed that included primary questions, follow-up inquiries, and probing questions. The guide comprised sections on participants’ understanding of the EDQS, perceived benefits and challenges, feasibility assessment, acceptability perspectives, and overall satisfaction. Interviews were conducted between August 18 and September 4, 2024.

The interview process started with greetings and an introduction, followed by the researcher clarifying the study’s aim, obtaining participants’ consent by approving the informed consent form, and initiating the recording. The interviewee’s background data and demographic information were subsequently gathered, followed by the predetermined interview questions and sections. Permission was obtained as well for the interview to be audio-recorded and for further utilization of the transcripts. Besides, the participants were informed that they had the right to withdraw from the study at any time.

The interview guide was validated through two preliminary interviews in which the researcher confirmed the suitability and consistency of the proposed questions but raised more focus on prob questions to deepen the answers. It is also validated through a group of healthcare quality experts, research supervisors, and experts from the World Health Organization (WHO), PMoH, and academia.

### Data analysis

The data from the ten interviews were analyzed using a manual thematic analysis approach. The analysis was primarily inductive, allowing themes to emerge from the data, and was subsequently interpreted through the lenses of feasibility and acceptability to enhance conceptual clarity.

Following each interview, the researcher documented reflections on the interview flow, initial impressions, covered and uncovered questions, interview dynamics, and general contextual information, including the date, time, organization, participant position, gender, and contact details. A unique code was assigned to each interview based on its order and the participating organization, and this code, along with the general information, was documented on each page of the transcript.

The researcher generates verbatim transcripts of each audio-recorded interview, including non-verbal expressions. The transcripts were read a few times line by line to familiarize himself with the participants’ perceptions and content of transcription, before starting the deep analysis [11].

Data analysis began with coding the transcribed interviews using a descriptive approach to identify meaningful units. This process involved labeling significant sentences or sections, highlighting them using different colors, and adding margin comments to assign codes corresponding to the highlighted text. These codes were linked to selected meaning units and iteratively refined during analysis [14, 15].

Related meaning units (Words, sentences, or paragraphs containing aspects related to each other through their content and context) are highlighted with the same color. Followed by the process of shortening while still preserving the core idea (condensed meaning unit). Themes and subthemes extracted from condensed meaning units [11, 14].

Thematic analysis for each interview was conducted manually by the researcher to identify themes and subthemes through a process of meaning condensation and structural analysis. A latent thematic analysis approach was applied to interpret the underlying meanings of the transcribed texts. To enhance credibility and analytical rigor, portions of the data analysis were independently reviewed by a peer or co-researcher to verify the selection of meaning units, subthemes, and themes [14].

The main themes and subthemes were summarized and examined in relation to the research question and the study context. The full dataset was then reread to ensure coherence, support a holistic understanding, and confirm the relevance and consistency of the identified themes [11]. Table 2 provides an illustrative example of thematic data analysis.

**Table 2:** Illustrative example of thematic data analysis

Meaning unit	Condensed Meaning unit	Code	Subtheme	Theme
I think implementing these standards is feasible and can seamlessly integrate into daily workflows.	EDQS integrates seamlessly into workflows.	Seamless integration	Workflow integration	Feasibility Enablers
Implementing EDQS is feasible and may improve health services for patients by reducing misdiagnoses in overloaded EDs.	EDQS can reduce misdiagnoses in overloaded EDs.	Feasibility and improvement	Workflow improvement	Benefits

## Results

### Main findings

**Fig. 2:**
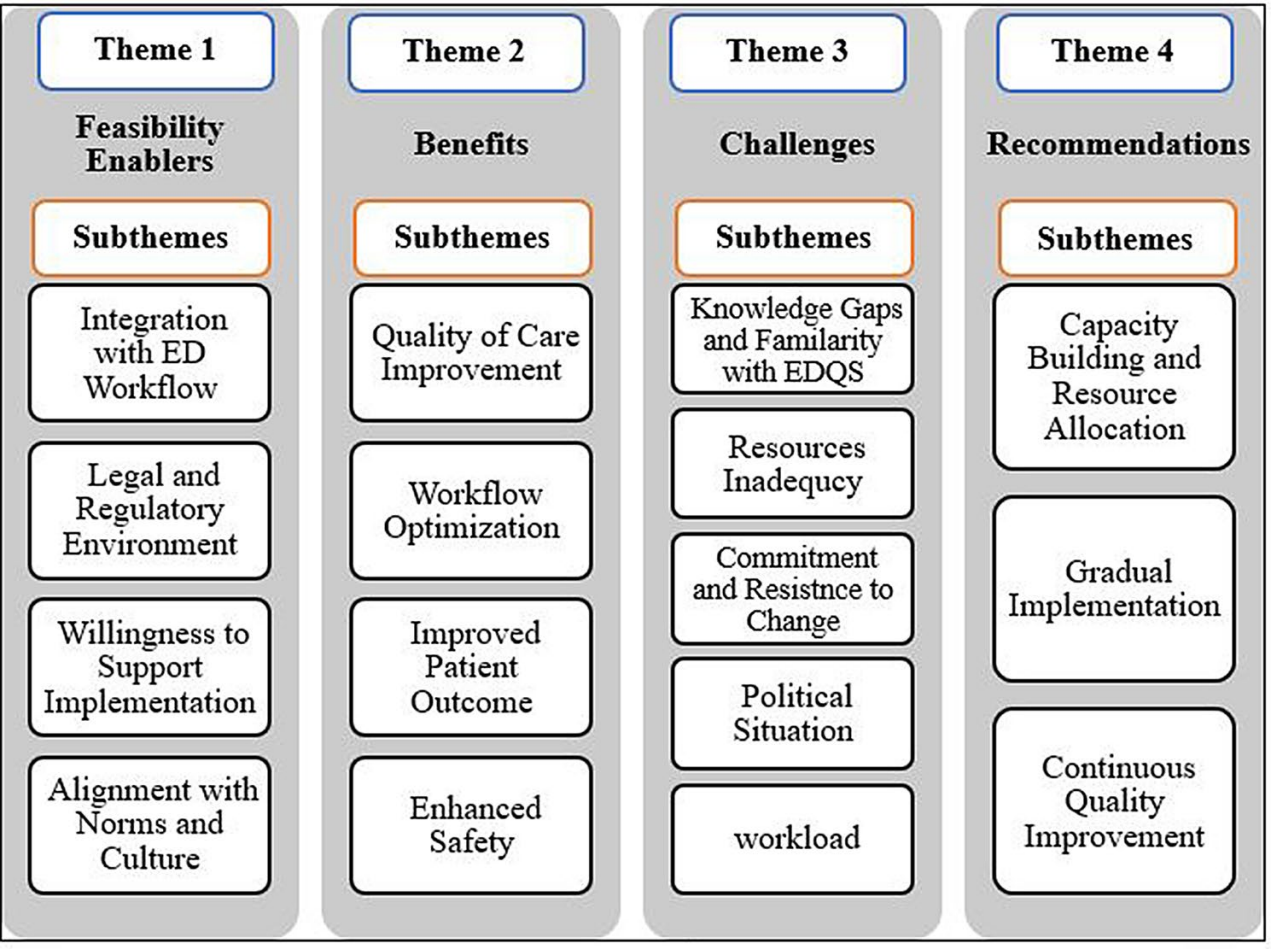
Summary of the main study findings’ themes and subthemes

The data analysis identified four key themes and 16 subthemes regarding the feasibility and acceptability of implementing EDQS in Palestinian EDs, as expressed by the interview respondents. These are summarized in Fig. 2, with illustrative quotes for each subtheme provided in Additional file 3.

#### Feasibility enablers

This theme explores the factors identified by participants as enabling the practical implementation of EDQS in Palestinian emergency departments, despite existing constraints.

##### Integration with ED workflow

This sub-theme reflects participants’ perceptions that the EDQS could be integrated into existing ED workflows, enhancing daily clinical practice without major disruption. The interviewees expressed confidence that EDQS would improve patient outcomes by reducing misdiagnosis and increasing consistency in care delivery, especially in overburdened EDs. One participant stated,

…, these standards are crucial and can be feasibly integrated into daily workflows, …. P4 P2L74

Many interviewees confirmed that EDQS would have a positive impact on the efficiency of EDs. In their views, integrating these standards is an opportunity to improve workflow rather than complicate it.

I believe these standards will impact positively and improve our workflow instead of hindering it…Thus, integrating these standards into the system is feasible. P2 P2L73

Some emphasized that the impact of these standards would be more evident once they were implemented and evaluated on the ground.

Based on my experience, implementing EDQS is feasible. This will become evident once we measure the impact of the implementation on relevant policies and procedures. For example, compliance with hand hygiene policies will demonstrate the results and feasibility after a period of implementation…. P3 P3L85

Most participants found the standards feasible and believed they could integrate them into current workflows with proper support. They noted that the EDQS aligned with existing systems, which could enhance care processes. Although some anticipated initial resistance to integration, they expected familiarity with the EDQS to grow over time, leading to routine use.

##### Legal and regulatory environment

This sub-theme highlights how participants perceived the legal and regulatory environment as supportive of quality improvement, fostering a positive context for EDQS implementation. Participants consistently highlighted the absence of any conflicting legal frameworks, with several noting that existing legislation, such as public health laws and medical waste management regulations, supports the implementation process. One participant indicated the absence of legal restrictions.

I believe there are no legal or regulatory barriers in Palestine against the implementation of EDQS. P1 P3L117

Others emphasized that current legislation is consistent with the EDQS, including essential legal frameworks like the Palestinian public health law.

I believe that there is no Palestinian law that contradicts or prevents the implementation of these standards, whether it is the public health law, the medical waste management law…. P6 P3L137

Participants emphasized that the laws not only prevent conflicts with the EDQS but also actively support improvement efforts.

Based on my background and experience all laws support the implementation of EDQS and have no conflict with them. P8 P3L130

Despite a favorable legal and regulatory environment, one participant highlighted that the successful implementation of EDQS relies more on leadership commitment and active engagement than on the legal framework.

…It can support improvement tools, but the key factor is leadership commitment and active engagement for effective implementation. P3 P3L125

##### Willingness to support implementation

This sub-theme emphasizes the strong dedication of frontline staff and leaders to support EDQS implementation, driven by a commitment to improving patient care over ideal working conditions. One participant stated that:

…, I personally accept these standards because they are for the benefit of both the patient and the doctor. P1 P3L127

One of the participants expressed his willingness to support the implementation of quality standards distinctively, saying,

On a personal level, I love quality and I love applying standards, so I am very ready to help apply and promote awareness about it…. P6 P4L161

The participants also emphasized the leadership’s willingness to support these standards and improvement initiatives, one stated,

As the head of the ED department, I am willing to support its implementation to the best of my ability. P10 P3L116

##### Alignment with norms and culture

This sub-theme indicates that participants perceived the EDQS as culturally compatible with existing professional norms and organizational values, which enhanced its acceptability within the Palestinian health system. As one participant noted:

From my experience, there is no conflict between these standards and the norms and culture of the health organizations and community; on the contrary, they are in line with the culture of improvement. P1 P4L150

Although participants generally felt no cultural hurdles, some did note that there may be infrequent sensitivity from patients or companions about specific aspects of EDQS, particularly related to triaging, as patients often feel their case is a priority. One participant explained:

There may be some sensitivity among patients and their companions to the issue of triaging patients in the triage department because every patient considers himself a priority,…. P3 P4L174

Participants emphasized that raising awareness among patients about the importance of these standards could address any concerns or misunderstandings. As one participant shared:

If the importance of applying the EDQS is explained to patients and their companions and raises awareness among them, and these standards become part of the system in the ED, I do not expect there to be any kind of sensitivity. P10 P4L143

#### Benefits of implementing EDQS

This theme reflects participants’ perceptions of the potential benefits of EDQS implementation, particularly in supporting more consistent, organized, and safer emergency care practices. The findings, categorized into four relevant subthemes, offer insights into how effective implementation of EDQS can enhance ED performance.

##### Quality of care improvement

This sub-theme demonstrates how participants perceived the EDQS to enhance emergency care by standardizing clinical processes. Participants emphasized that EDQS can potentially improve care quality and service delivery in EDs, resulting in better outcomes and increased efficiency. One participant noted that,

There are numerous benefits to implementing EDQS. In general, it improves the quality of services in the EDs, especially regarding safety issues and clinical practices. P3 P2L63

##### Workflow optimization

This sub-theme reflects participants’ views on EDQS’s potential to improve emergency department workflow, task prioritization, and patient flow. The following statements reflect the perspectives of the respondents regarding these findings.

Proper management and timely access to patients and their companions are facilitated, as well as the prioritization of cases and waiting through triage standards. P3 P2L69

##### Improved patient outcome

This sub-theme highlights participants’ expectations that EDQS implementation could enhance patient outcomes through timely, coordinated, and evidence-based care. A Participant indicated that,

The primary expected benefit of implementing EDQS is the reduction of mortality rates in EDs due to malpractice. A secondary benefit is the establishment of standardized procedures among staff, leading to consistent practices across all EDs that adopt the EDQS. P6 P2L69

##### Enhanced safety

This sub-theme participants perceived that compliance with safety measures may protect the staff and patients from harm, fostering a more secure healthcare environment and decreasing preventable errors. Quotes from the interviewed doctors and nurses support this.

…, When enforced, they benefit both patients and doctors…, These standards protect the doctor by providing documented evidence, such as consent forms…. P1 P2L66-67

#### Challenges of implementing EDQS

This theme captures the key challenges that participants perceived as constraining the implementation and sustainability of EDQS in Palestinian EDs.

##### Knowledge gaps and familiarity with EDQS

This sub-theme reflects participants’ limited and uneven familiarity with EDQS, highlighting the need for structured and ongoing training to support effective implementation. One participant stated,

I briefly learned about EDQS during a training session with the researcher and the hospital’s quality and patient safety department. P4 P1L5

Participants showed awareness of some aspects of EDQS, like triage protocols and patient safety; however, they often revealed only a superficial understanding of the standards relevant to their roles. One participant said that

The standards address patient handling in the ED, including triaging and preparing patients for admission. P10 P1L38

Within the same context of knowledge, several respondents indicated possessing limited experience with certain international standards. Some interviewees remarked that:

I am familiar with standards such as clinical and administrative standards. I am knowledgeable in performing CPR for arrested patients according to protocols, basic life support, advanced life support, coverage of the ED with needed staff around the clock, and training the staff. I also had a short session explaining EDQS standards’ structure and requirements. P3 P2L44

There were notable gaps in understanding the critical areas of infection control and patient safety, which are both crucial components of EDQS. Although respondents had witnessed colleagues in the quality departments executing infection prevention protocols, they frequently lacked direct, practical experience.

In addition, the participants showed a lack of formal training in the practical implementation of the Patient Safety Friendly Hospital Initiative [16], despite their seeming lack of awareness of it.

##### Resources inadequacy (Inadequate financial, human, and physical resources)

This sub-theme illustrates how shortages in financial, human, and physical resources were perceived as major constraints affecting the depth and sustainability of EDQS implementation, as noted by interview participants.

Training resources are limited internally and reliant on external funding and projects. P2 P3L86

In addition, the financial crisis that is sweeping Palestine is affecting the infrastructure upgrades necessary to meet the health service quality standards, such as the redesign of triage and waiting areas, or areas that do not comply with the requirements of the standards, as one participant stated.

Palestine faces major challenges, especially financial ones that affect all sectors, especially healthcare. These issues limit training and infrastructure in EDs…. P3 P3L102

Participants emphasized that implementing some standards may not require costs, while others related to infrastructure specifically and redesign are associated with additional costs, as some of the participants said:

…There may be additional costs to make some changes to the infrastructure as requirements for some standards, like redesigning for entrance, exit, triage, waiting area, or safety issues. P4 P3L93

Several participants believed that implementing the EDQS could reduce unnecessary resource use and improve clinical organization.

…Implementing infrastructure standards may incur short-term costs, but long-term savings… P6 P3L108

…These benefits can be categorized into short-term advantages for patients and healthcare providers, and long-term benefits for the community, including lower mortality and morbidity rates and better resource management. P6 P2L69

Participants noted that resources are centrally allocated in PMoH hospitals, which supports overall management but may limit local flexibility, while decentralized models were perceived as better enabling hospitals to adapt to local needs and meet standards.

The central budget seems unfair to some hospitals due to varying capacities and workloads. A decentralized budget could enhance quality improvement initiatives and make it easier for hospitals to manage their needs…. P6 P3L102

The analysis of interview data revealed that the infrastructure limitations and shortage of ED staff were frequently cited as barriers to implementing EDQS. As quoted from the responses below.

The first challenge is often inadequate infrastructure, such as designated areas for CPR, triage, patient and staff waiting, and hand hygiene, which can result from poor design and construction. The second challenge includes staff shortages, …. P4 P2L65

Despite the existence of an EM residency program and the increase in the number of EM specialists in Palestine, EDs still suffer from a shortage of EM specialists, in addition to nursing and allied health professionals, according to the participants’ point of view, as most of them confirmed this.

…Additionally, there is a shortage of emergency specialists. P10 P3L94

Equipment availability in EDs varies. Most respondents indicated their departments are well-equipped and meet EDQS standards, though some reported challenges with equipment resources and quality. One participant noted:

The essential equipment for ED in our hospital is available, like, monitors, DC shock, ultrasound, and camera laryngoscope. I think in this regard it is good and aligned with EDQS. P9 P3L103

Some respondents identified specific gaps and variability in technological resources among hospitals. Although ultrasound machines are typically accessible, concerns regarding the quality of certain provided items persist. One participant noted:

There are some shortages in equipment, such as ultrasound, but they now provide us with low-quality portable ones. The X-ray machine is far away from the department, which does not align with the required standards and may be a constraint. P1 P3L111

##### Commitment and resistance to change

This sub-theme demonstrates how resistance to change, often linked to workload pressure and uncertainty about sustained support, was perceived as a challenge to consistent engagement with EDQS.

Interviewees said that a lack of real commitment and collaboration from staff could hinder the implementation phase, with some emphasizing the importance of serious commitment.

The concerns arise from serious commitment and collaboration between staff. P1 P4L133

While others pointed out another issue that might hinder implementation, which is resistance to change among technical or administrative cadres. One of them stated,

Cultural resistance to change, …, hinders EDQS implementation. P9 P2L69

Sustainability of implementation was frequently raised by participants as a concern. Several participants linked these concerns to organizational continuity, particularly leadership turnover and shifting strategic priorities. One participant stated:

Concerns about the sustainability of the implementation and the systematic monitoring of compliance with these standards arise from leadership turnover and strategic changes. P9 P3L123

One responder indicates an optimistic attitude toward the viability of EDQS implementation by expressing confidence that there are no significant obstacles.

I don’t think there is a significant concern in implementing the EDQS. P10 P3L120

##### Political situation

This sub-theme captures the impact of political instability and financial constraints on the health system, which participants viewed as external factors threatening the continuity of EDQS implementation. The interviewee stated.

Challenges can arise at the national level, including political situations, military attacks by occupying forces, and financial crises…. P6 P2L78

#### Workload

This sub-theme highlights how high patient volumes and staffing pressures limited the time and capacity available for staff to engage with new standards and implementation activities. Many respondents state concern about the additional workload from implementing the EDQS, especially in EDs, where staff are already overwhelmed. Extra tasks, such as documentation and administrative processes, add to this burden.

Concerns primarily arise from the workload in EDs, the additional effort required for standard implementation, and the accompanying documentation process. P3 P3L147

Ultimately, the results of the interview analysis showed that participants believed that EDQS is feasible, acceptable could be implemented, regardless of various challenges, whether related to resources or otherwise reasons. Some participants said:

In my opinion, whatever the reasons or challenges, this should not conflict with or affect the feasibility of the implementation. Even if there is a lack of resources or infrastructure…. P5 P2L82

Despite the challenges of the current political situation, such as attacks on health facilities and logistical challenges in providing resources, participants stressed that essential services and life-saving care provided by EDs must receive attention to improve quality. One participant said

The current situation may delay or make it unfeasible to implement due to attacks on healthcare facilities, …As a result, our priorities should focus on providing essential services and life-saving emergency care. P3 P3L93

One participant mentioned an example that emphasized the feasibility of implementing the standards despite potential challenges, which was that if the computerized systems used to document a patient’s medical record failed, it was possible to switch to a manual system, ensuring compliance with relevant standards.

I believe these standards can be feasible despite potential challenges. We can document and manage medications safely, even if limited resources affect compliance. If the computerized health information system fails, we can switch to a manual system, but documentation must persist and comply with relevant standards. So, I believe no reason could make implementing EDQS unfeasible. P8 P3L104

The overall interviewee agrees that no valid excuses make EDQS unfeasible while logistical and infrastructural barriers exist.

#### Recommendations

This theme summarizes participants’ recommendations for supporting feasible and sustainable implementation of EDQS, emphasizing the need for system-level support, phased approaches, and continuous learning. These recommendations are categorized into three subthemes, as follows:

##### Capacity building and resource allocation

This sub-theme highlights participants’ emphasis on strengthening workforce capacity and ensuring adequate financial and material resources as foundational requirements for EDQS implementation.

Training is one of the most critical components for capacity building and the successful implementation of the EDQS. The workforce needs to be prepared through specialized induction and advanced training, ensuring that they are familiar with new protocols and quality standards. Effective training on standard implementation is crucial in helping staff transition smoothly to the new system.

Numerous participants emphasized the need for creating customized training programs to ensure that all personnel understand and implement EDQS according to their respective duties. One participant stated.

…Develop a training program that aligns with the specific duties of each health profession…. P3 P4L131

Resource allocation, addressing gaps in human resources, and ensuring that the infrastructure meets EDQS requirements were highlighted by the respondents.

Filling the gap in human resources and redesigning the infrastructure to match the requirements of the standards. P5 P3L110

Respondents indicate that centralized budgeting provides predictable resource flow, while flexibility and decentralization enable departments to address patient needs effectively. *“The central budget seems unfair to some hospitals due to varying capacities and workloads. A decentralized budget could enhance quality improvement initiatives and make it easier for hospitals to manage their needs…”. P6 P3L102.*

##### Gradual implementation

This sub-theme reflects participants’ recommendations for a phased and flexible implementation approach that allows adaptation to local constraints and incremental capacity development. While acknowledging existing challenges such as limited infrastructure and resource constraints, many participants noted that these barriers should not prevent the gradual and partial integration of standards. They recommended adopting EDQS in phases at the ED level and gradually expanding it at the national level, allowing for adjustments and improvements as resources permit. As follows

Despite the challenges, these standards are crucial and can be feasibly integrated into daily workflows, even if their implementation is gradual and partial. P4 P2L74

Participants described expectations that the EDQS could be gradually integrated into EDs over time. Some participants anticipated initial resistance to implementation but suggested that increased familiarity could support incorporation into routine practice. One participant stated,

…, initial resistance may occur, but it will gradually become integrated into the system and daily workflow of the ED in Palestine. P10 P2L72

##### Continuous quality improvement

This sub-theme captures participants’ recognition of ongoing monitoring, feedback, and learning processes as essential to sustaining EDQS implementation over time. As confirmed by the respondent.

…, sustainability of the implementation and the systematic monitoring of compliance are crucial for improvement and sustainability. P9 P3L123

## Discussion

### Feasibility enablers and alignment with legal and cultural context

This study explored the feasibility and acceptability of implementing Emergency EDQS in Palestinian EDs, focusing on the perspectives of frontline staff. Participants noted that integrating EDQS into daily workflows was feasible, not because conditions were ideal, but because the standards offered practical tools for improving coherence and prioritization in a fragmented care environment. Feasibility was defined in relation to existing constraints rather than ideal emergency care benchmarks.

While studies from other low-resource settings, like those by Goenka, et al. (2024), have shown improvements in emergency ED performance with quality interventions, the perceived feasibility in the Palestinian context is different. Participants’ views are influenced by their prolonged exposure to systemic challenges, leading them to value standardized guidance for supporting decision-making and enhancing accountability under pressure. In conflict-affected health systems, quality standards are seen more as stabilizing frameworks that promote resilience and ethical practice, rather than just performance-enhancing tools. Thus, the perceived feasibility in this study should be seen as an indicator of readiness for implementation rather than proof of actual performance improvement [17].

Participants indicated that there were no perceived legal or regulatory barriers to implementing Emergency Department Quality Standards (EDQS) in Palestine. However, this should be interpreted with caution and not seen as evidence of complete regulatory readiness. The lack of reported obstacles may reflect regulatory flexibility and an absence of enforceable emergency department quality legislation rather than a comprehensive governance framework.

While the overall legal framework is supportive of quality improvement initiatives for patient safety and access to services, this support is more general and not specifically tied to the ED. Unlike other contexts where regulatory requirements motivate health quality standards, Palestine’s situation suggests that implementation is enabled by regulatory gaps instead of formal endorsement [18].

Additionally, although World Health Organization guidelines stress the accountability of healthcare providers to meet quality standards, relying on professional accountability without enforceable regulations places a heavy burden on frontline staff [19]. This situation illustrates that while regulatory flexibility can facilitate initial efforts, long-term sustainability will require formal policy integration and accountability mechanisms that support healthcare workers’ commitment.

Participants generally expressed their acceptance of the EDQS and their willingness to implement them, driven by a perceived commitment from leadership, even though there were identified knowledge gaps. In the Palestinian context, this willingness reflects a professional resilience that has developed amidst ongoing resource constraints and political instability, rather than an undisputed readiness for implementation [20]. The EDQS were regarded as helpful tools that could assist staff in providing safer care under pressure.

Previous studies, such as those by Kelly, et al. (2023), have highlighted that staff engagement and leadership support are crucial for implementing quality standards [21]. However, this study emphasizes that in conflict-affected settings, these factors also act as ethical and motivational mechanisms that sustain practice, even in the absence of robust system-level support. Therefore, implementation efforts should align this commitment with realistic capacity-building strategies and phased support.

Participants consistently regarded the EDQS as culturally acceptable and compatible with existing institutional and societal norms within Palestinian health organizations. Rather than reflecting a lack of cultural sensitivity, this finding suggests a shared professional culture where quality improvement initiatives are seen as ethically aligned with patient safety and service equity, especially in the context of prolonged system strain. While some studies in other settings report cultural resistance to externally derived standards, the situation in Palestine indicates a pragmatic approach to improvement initiatives that are viewed as supportive of frontline practice. This alignment with local culture enhances the perceived feasibility of integrating EDQS, highlighting the importance of contextual adaptation rather than assuming cultural neutrality [21].

This finding is supported by evidence from Palestinian healthcare settings showing that work environment characteristics, including leadership support and organizational context, are strongly associated with professional quality of life, burnout, and staff engagement [20].

### Expected benefits of implementing EDQS

The EDQS was perceived by participants as a way to improve consistency and structure in emergency care, especially within a resource-limited Palestinian ED. While previous research suggests quality standards can improve health outcomes, these findings reflect anticipated, rather than empirically proven, benefits. Therefore, expected performance improvements should be interpreted with caution and require evaluation through pilot studies [22].

Optimizing ED workflows through structured standards was seen by participants as a potential way to improve patient management and reduce waiting times. Although previous studies, such as those by Kaushik et al., have shown the benefits of workflow optimization in specific ED processes [23], the current findings reflect expectations in a highly constrained environment. In this context, standardization is valued as a tool to enhance patient flow and prioritize tasks during varying levels of demand, rather than as proof of actual performance improvements [24].

Additionally, implementing EDQS may foster a safer environment for both patients and healthcare providers, consistent with the WHO’s patient safety-friendly hospital framework [16]. A recent study by Mehreen, et al. (2024) further supports that adherence to standards, such as international patient safety goals, enhances safety culture in EDs, reduces medical errors over time, and improves overall quality [25].

### Challenges to EDQS implementation

This study reveals a knowledge gap regarding EDQS among frontline staff in Palestinian EDs. While some participants demonstrated a basic understanding of standards like patient safety and triage, their overall familiarity with EDQS was limited. These findings align with previous studies indicating that knowledge deficits hinder the implementation of quality standards, particularly in low-resource healthcare settings. In the Palestinian context, these gaps appear to reflect limited exposure to structured training rather than resistance to standards. This finding underscores the need for regular, context-adapted training as a prerequisite for feasible and sustainable EDQS implementation [26, 27]. Previous studies recommend embedding quality improvement approaches like EDQS within routine professional development to address such gaps [28].

Experience with relevant standards emphasizes the challenges associated with integrating EDQS into routine practices. Despite familiarity with international standards such as BLS, ALS, and the WHO’s Patient Safety Friendly Hospital Initiative, the absence of practical implementation highlights significant limitations in training and infrastructure. ED personnel need continuous training programs to effectively implement these EDQS in practical contexts, thereby enhancing their understanding of the standards. This is aligned with previous studies that confirm the need for regular training and capacity building for human resources and face-to-face interaction [29].

Participants found the EDQS to be implementable without significant conflict, as they aligned well with existing procedures and protocols in EDs. However, limitations related to infrastructure and staff training were identified as potential barriers to full compliance. While some studies indicate that quality standards can be cost-effective in the long run, the Palestinian context emphasizes the need for initial investments in infrastructure, workforce development, and implementation support. These investments are crucial for translating alignment into sustained practice, especially in resource-constrained and unstable environments [29, 30].

Participants identified a lack of training, staff shortages, limited leadership commitment, and inadequate infrastructure as key challenges that may hinder compliance with quality standards in Palestinian EDs. While similar barriers have been reported in other low-income settings, including by Oleribe, et al. (2019), these challenges in Palestine appear to be compounded by chronic system strain, underscoring the need for context-specific capacity building, leadership engagement, and sustained investment rather than standalone training interventions [26].

Respondents identified resistance to change as a significant challenge in implementing EDQS. While resistance is a common theme in quality improvement literature, in the Palestinian emergency care context, it seems to be closely linked to factors such as workload pressure, limited previous exposure to structured standards, and uncertainty about ongoing support rather than opposition to improvement itself. This highlights the need for facilitation strategies that focus on staff involvement, trust-building, motivation, and flexible implementation approaches to ensure the sustainable adoption of EDQS [31].

Participants noted that a significant challenge to sustaining the implementation of EDQS is the lack of structured follow-up and evaluation mechanisms. In the Palestinian context, the limited routine performance monitoring hinders the feedback and accountability necessary for ongoing improvement. Previous studies, such as those by Schmutz et al., highlight the importance of continuous monitoring through multidisciplinary approaches. These findings emphasize the need for tailored monitoring systems that support compliance and learning during the implementation of EDQS [32].

The political situation and instability in Palestine, because of the Israeli occupation and the resulting financial crisis, affect all aspects of life, including the health sector in general and EDs in particular. This is what the study showed as one of the challenges that threaten the implementation of the EDQS and its sustainability. This is highlighted in the United Nations report on the economic situation in Palestine, as this report confirms the financial challenges that lead to a shortage of resources, the inability of the health system to provide health services, and its inability to develop and grow or implement improvement initiatives, including the implementation of EDQS [33].

The study found that the increased workload and high utilization of ED services for non-emergency cases are one of the challenges, likely resulting from a lack of awareness about the ED’s role among patients. Raising this awareness is essential for implementing an effective emergency services quality system. This is in line with other studies such as Alotaibi, et al. (2023) and Abuljadail, et al. (2024), and the need to develop solutions and interventions to improve utilization, including public awareness programs and directing them to primary health care centers instead of EDs [34, 35]. Suggesting that increasing public engagement and education could help support quality improvement efforts and minimize the workload in the EDs.

Interview findings should not be interpreted as indicating a lack of implementation challenges; instead, they reflect a strong sense of professional resilience among ED staff. In the Palestinian context, the willingness to pursue the implementation of EDQS, despite infrastructural, workforce, and political constraints, seems to stem from ethical commitment and adaptive leadership rather than favorable circumstances. This interpretation aligns with the International Federation of Emergency Medicine (IFEM) framework, which emphasizes that while implementation challenges in emergency care can be significant, they are often counterbalanced by enabling factors, notably effective leadership. As highlighted in the IFEM framework, sustained quality improvement is facilitated by leaders who actively promote supportive environments and remain engaged in addressing care gaps. This underscores the critical role of leadership in transforming resilience into practical and sustainable implementation [6].

Interview findings indicated that EDQS was perceived as feasible and applicable, with participants highlighting that the availability of qualified personnel, essential equipment, and a generally supportive legal environment were enabling factors. However, in the Palestinian context, these elements were viewed as sufficient to initiate implementation rather than as guarantees of sustained compliance. Consistent with prior studies, successful quality improvement initiatives require a combination of workforce capacity, appropriate infrastructure, leadership commitment, continuous monitoring, and financial and political stability, underscoring the need to interpret perceived feasibility alongside broader system conditions [6].

Moreover, previous studies highlight the need for developing effective and sustainable strategies that align with potential challenges to address issues related to these enabling elements [36]. However, to gain a deeper understanding of the challenges, real feedback should be gathered through the implementation of the standards. This feedback will inform the development of appropriate strategies and interventions to tackle any identified challenges.

Participants expressed concerns about accepting the standards, mainly due to the potential for an increased workload, particularly regarding documentation requirements. These concerns align with findings from previous studies, which suggest that organizational leadership should acknowledge employees’ efforts and take their time and workload into account. By doing so, leaders can enhance employee performance and foster a greater acceptance of the standards [21, 36].

The study identified several key factors that facilitate the acceptance of standards within the EDs. Respondents highlighted the importance of support and guidance from management and leadership, as well as the need for collaboration with external institutions involved in emergency services. Given the challenging circumstances and limited resources in Palestine, external support proves essential for implementing these standards; it provides necessary resources and training.

Leadership plays a crucial role in directing and leading the implementation process, including follow-up and evaluation, to ensure successful adherence to these standards and continuous quality improvement. The study titled “Best Practices in Management and Leadership” by Enahoro, et al. (2024) emphasized the interactive role of effective leadership in promoting quality initiatives in healthcare. It categorizes different leadership styles, noting that transformational leadership boosts motivation and participation, service leadership fosters teamwork and shared responsibility, and democratic leadership encourages comprehensive decision-making [37]. This agrees with the study’s interviewees.

As for the impact of external support, including collaboration and partnership on acceptance of EDQS implementation, this is consistent with the respondents’ opinion that it is a positive factor for the sustainability of the implementation, improving performance, raising awareness, and exchanging knowledge and experiences [36].

### Recommendations for supporting EDQS implementation

Successful EDQS implementation requires targeted system-level support, not just adherence to standards. Participants stressed the need to strengthen workforce capacity and allocate sufficient resources to address knowledge gaps and operational constraints. Capacity building should include structured training and practical support, integrating clinical and administrative aspects of the standards.

Participants recommended a gradual, phased implementation to allow adaptation to local conditions, minimize disruption to emergency services, and reduce resistance to change, especially in high-demand, resource-constrained emergency care settings.

Finally, embedding EDQS in continuous quality improvement is essential for sustainability. Consistent monitoring, feedback, and learning are critical for reinforcing adherence, fostering ongoing improvement, and adapting to evolving system challenges.

## Limitation

This study has several important limitations that should be considered when interpreting the findings. First, the analysis focused on perceptions of feasibility and acceptability rather than on the direct implementation of the EDQS in routine practice. Consequently, the results reflect early, perception-based implementation outcomes and do not provide evidence of actual feasibility, fidelity, or effectiveness.

Second, the small sample size (*n* = 10) was appropriate for achieving in-depth qualitative insights and thematic saturation, but it limits the transferability of the findings beyond the study context. Third, participants were drawn exclusively from emergency departments in the West Bank; therefore, the findings may not be applicable to the Gaza Strip, where healthcare delivery occurs under markedly different and highly constrained conditions.

Fourth, the first author’s dual role as both researcher and developer of the EDQS may have introduced social desirability or confirmation bias, potentially influencing participants’ expressed views on acceptability and implementation. Although steps were taken to reduce this risk—such as voluntary participation, assurances of confidentiality, and reflexive analysis—this influence cannot be entirely excluded.

In addition, most interviews were conducted remotely due to contextual constraints, which may have limited rapport and the depth of interaction compared with in-person interviews. Finally, participants’ limited prior training and exposure to the EDQS may have shaped their interpretations of the standards and contributed to variation in their responses.

### Implications of this study

At the policy level, the findings highlight the need for the PMoH to formally integrate the EDQS within national emergency care and quality governance frameworks. Clear policies, regulatory endorsement, and aligned implementation strategies are required to translate perceived feasibility into sustained practice. Policymakers should also prioritize resource allocation, leadership accountability, and coordination mechanisms to support phased and context-adapted implementation of EDQS across EDs.

For hospital and ED leaders, the findings underscore the importance of leadership engagement, workforce capacity building, and realistic implementation planning. Given existing resource and workload constraints, EDQS implementation should be gradual and flexible, supported by structured training, protected time for staff engagement, and simple monitoring mechanisms. Leadership at the facility level plays a critical role in fostering staff motivation, addressing resistance to change, and ensuring that quality initiatives are aligned with daily clinical workflows.

This study assessed the perceived feasibility and acceptability of EDQS rather than their impact on healthcare delivery. Future research should therefore focus on pilot implementation studies to test the standards in practice, beginning with baseline assessments of ED capacity and performance. Mixed-methods and longitudinal evaluations are needed to examine adoption, fidelity, and sustainability, as well as to assess the effects of EDQS on patient outcomes, safety, and service efficiency. Findings from such studies can inform iterative refinement of the standards and guide decisions on a broader scale-up.

## Conclusion

This study demonstrates that frontline ED staff perceive the EDQS as both feasible and acceptable within the Palestinian context, despite persistent challenges related to knowledge gaps, resource constraints, and system pressures. Implementation is most likely to succeed when enabling factors, particularly leadership commitment, targeted training, and adequate resource support, are actively addressed. A phased implementation approach, accompanied by continuous monitoring and evaluation, may help translate perceived feasibility into routine practice and support incremental improvements in emergency care quality.

## Supplementary Information

Below is the link to the electronic supplementary material.

Supplementary Material 1

Supplementary Material 2

## Data Availability

All data generated or analyzed during this study are included in this paper and its supplementary information files. Additional supporting data are available from the corresponding author upon reasonable request.

## Abbreviations

EDQS: Emergency Department Quality Standards
ED: Emergency Department
EM: Emergency Medicine
WHO: World Health Organization
COREQ: Consolidated Criteria for Reporting Qualitative Research
HREC: Human Research Ethics Committee
MoH: Ministry of Health
PMoH: Palestinian Ministry of Health
QPS: Quality and Patient Safety
MD: Medical Doctor
JCI: Joint Commission International
ALS: Advance Life Support
BLS: Basic Life Support
CPR: cardiopulmonary resuscitation
ACLS: Advanced Cardiac Life Support
IFEM: International Federation of Emergency Medicine
