# Supplementary material for: Assessing the feasibility and acceptability of implementing emergency department quality standards in Palestine: a qualitative study
Source: BMC Health Serv Res. 2026 Feb 24;26:510. doi: 10.1186/s12913-026-14199-6 (PMC13078010; doi:10.1186/s12913-026-14199-6)
Supplement: Supplementary file 1 — Supplementary Material 1 [file 12913_2026_14199_MOESM1_ESM.pdf]

Additional file 2: Interview guide for assessing the feasibility and acceptability of Emergency Department Quality Standards (EDQS) among experienced emergency department staff:

Title of Study: Feasibility and Acceptability Study for Implementing Emergency Department Quality Standards (EDQS). **This aim of ethics application.**

in

Developing Contextual Quality Standards for Emergency Departments in Palestine

Researcher: Abed Alra'oof Mohammad Saleem Bani Odeh, MCLS, PhD candidate - Emergency Medicine in the Faculty of Health Sciences at the University of Cape Town.

Supervisor: Dr. Willem Stassen

Co-Supervisor: Professor Motasem Hamdan

Co-Supervisor: Professor Lee Wallis

Field of Research: Emergency Medicine

Introduction:

- Begin with a warm introduction and reassuring confidentiality.
- Confirm the participant's consent for audio recording.

- Briefly explain the purpose of the interview:

"We are conducting a pilot study to assess the feasibility and acceptability of implementing Emergency Department Quality Standards (EDQS) in emergency departments. Your insights as an experienced staff member are invaluable for this study".

  - o Can you please tell me about your role and responsibilities in the emergency department?
  - o How long have you been working in this department?
  - o Other issues to consider are gender and educational background (GP, emergency physician, nurse, etc.).

1. **Section 1: Understanding of EDQS**

    1.1. Have you heard about the EDQS before?

    1.2. Can you describe your understanding of what EDQS is?

    1.3. Could you please let me know how familiar you are with EDQS and its associated domains and subdomains? Have you ever had any training in this regard?

    1.4. Have you had any previous experience with similar quality standards in emergency departments?

    1.5. How do you think EDQS aligns with current practices and protocols in the emergency department?

2. **Section 2: Perceived Benefits and Challenges**

    2.1. What are the potential benefits of implementing EDQS in an emergency department from your point of view? Can you give me examples?

    2.2. From your perspective, What challenges or obstacles do you expect in the implementation of EDQS? Can you please give me examples?

3. **Section 3: Feasibility Assessment**

    3.1. In your opinion, how feasible is it to integrate EDQS into our daily workflow?

    3.2. Is there any reason that could make implementing EDQS unfeasible, such as resource limitations, logistical issues, or operational challenges?

        3.2.1. How would you evaluate the availability of financial resources, taking into account budget considerations for training, infrastructure improvements, and any potential additional costs associated with implementing the standard?

        3.2.2. How would you evaluate the availability of qualified staff in your ED? Do you think additional training or hiring is necessary? What type of training or in what areas?

        3.2.3. To what extent is the technological infrastructure and equipment in EDs available and aligned with the requirements of EDQS?

3.2.4. Do you believe that there are any legal or regulatory barriers to implementing the EDQS and identifying the steps needed for compliance? Explain?

3.3. What are the key factors or recommendations that would enable successful integration of EDQS into daily operations, in your opinion?

4. **Section 4: Acceptability and Stakeholder Perspectives**

4.1. To what extent do you and your colleagues in the emergency department accept the EDQS?

4.1.1. How willing are you to adopt and implement the EDQS?

4.2. Are there any concerns or reservations that you think your colleagues might have regarding EDQS?

4.3. How can be enhanced the acceptance of EDQS among the emergency department staff?

4.3.1. How would you describe the commitment of hospital administrators and ED to implementing the EDQS from your perspective?

4.3.2. Do you think that partnering with relevant healthcare organizations, governmental bodies, or international agencies can assist and guide the implementation of EDQS? If so, how?

4.4. How well do you think the EDQS aligns with the cultural norms and practices of our organization?

4.4.1. In your opinion, how culturally sensitive is the EDQS?

5. **Section 5: Suggestions for Improvement and overall satisfaction**

5.1. Based on your understanding and experience, do you have any suggestions for improving or refining EDQS? or modification to enhance its effectiveness?

5.2. On a scale from 1 to 5, how satisfied are you with the EDQS: 1 (Not satisfied at all) to 5 (Very satisfied)

Closing and conclusion:

- Is there anything else you would like to add or discuss regarding EDQS and its potential implementation in our emergency department?

- Thank you for your time and valuable insights during this interview.

Note: The semi-structured nature of the interview guide allows for flexibility in probing and exploring unexpected topics that may arise during the conversation. Listening actively and encouraging participants to share their experiences and perspectives openly is important.
