# Supplementary material for: Assessing the feasibility and acceptability of implementing emergency department quality standards in Palestine: a qualitative study
Source: BMC Health Serv Res. 2026 Feb 24;26:510. doi: 10.1186/s12913-026-14199-6 (PMC13078010; doi:10.1186/s12913-026-14199-6)
Supplement: Supplementary file 2 — Supplementary Material 2 [file 12913_2026_14199_MOESM2_ESM.pdf]

Additional file 3: *Illustrative quotes for subthemes.*

Title of Study: Feasibility and Acceptability Study for Implementing Emergency Department Quality Standards (EDQS). **This aim of ethics application.**

in

Developing Contextual Quality Standards for Emergency Departments in Palestine

Researcher: Abed Alra'oof Mohammad Saleem Bani Odeh, MCLS, PhD candidate -Emergency Medicine in the Faculty of Health Sciences at the University of Cape Town.

Supervisor: Dr. Willem Stassen

Co-Supervisor: Professor Motasem Hamdan

Co-Supervisor: Professor Lee Wallis

Field of Research: Emergency Medicine

Theme	Subtheme	Example on Illustrative Quotes
1. Feasibility Enablers	1.1. Integration with ED workflow	*"…the standards are applicable, feasible, and easy to integrate into the daily workflow, …". P5 P2L77.*
		"I think implementing these standards is both feasible and can seamlessly integrate into daily workflows". P7 P2L85.
		"It is feasible; initial resistance may occur, but it will gradually become integrated into the system and daily workflow of the ED in Palestine". P10 P2L72.
	1.2. Legal and Regulatory Environment	*"To my knowledge, there are no Palestinian laws that conflict with the implementation of these standards". P4 P3L112*
		"Palestinian law aligns with these standards, including Palestinian public health law". P7 P3L119.
		"I believe the Palestinian laws facilitate the implementation of EDQS and improvements and have no conflict with them". P10 P3L104.
	1.3. Willingness to support implementation	*"as I mentioned at the beginning anything could contribute to improving the EDs services all of us will support as well as accept…". P2 P3L110.*
		"I am willing and fully support the implementation of these standard…". P3 P3L143.
		"we are willing and eager for implementation and support of the EDQS. because will simplify and organize the workflow". P7 P3L133.

	1.4. Alignment with Norms and Culture	*"...I think it fits very well with the culture and norms of both the community and the organizational level and nothing is conflicting". P6 P4L203.*
		"To my knowledge, there are no Palestinian laws that conflict with the application of these standards". P4 P3L112.
**2.** Benefits of Implementing EDQS	2.1. Quality of Care Improvement	*"..., Key benefits include enhanced patient satisfaction, improved performance, fewer medical errors, a positive hospital reputation, reduced infections, and shorter waiting times" P2 P2L60.*
	2.2. Workflow Optimization	*"Potential benefits include shorter patient stays in the ED through effective triaging and prioritization of urgent cases" P7 P2L68.*
		"organize the ED and patient flow and reduce patient waiting". P8 P2L74.
	2.3. Improved Patient Outcome	*"..., improves treatment outcomes, and decreases complications and mortality-morbidity rates through timely intervention in emergencies". P7 P2L71.*
	2.4. Enhanced Safety	*"..., the standards related to ED infrastructure and design affect the quality and safety of services provided to emergency patients…". P3 P2L67.*
		" ...EDQS enhanced patient safety, staff protection, reduced medical errors, and minimized patient harm and complication". P10 P2L60.
	3.1. Knowledge Gaps and Familiarity with EDQS	*"I have some familiarity with the standards, but I need to explore their requirements further as the training was brief and general. P2 P2L45.*

3. Challenges of Implementing EDQS		*"I have some experience in this area by observing colleagues in the hospital's quality department and their efforts to implement patient safety and infection control standards". P9 P2L50.*
		"I have little knowledge about hospital quality and WHO patient safety standards, including infection prevention and control". P5 P2L49.
	3.2. Resources inadequacy	*"Certainly, the availability of resources for implementing the standards is a challenge. Like many other hospitals, we encounter difficulties in providing specialized training, such as basic and advanced life support for nurses and doctors, due to limited resources, as well as meeting other standards related to infrastructure improvement…". P1 P3L96.*
		"…The additional costs I think are more related to redesigning the infrastructure to meet the standards requirements if the infrastructure is not suitable". P5 P3L9.
		"As a hospital affiliated with the Ministry of Health, most of the human and material resources are available centrally. Even if there are some of these resources, we work to provide health services in the EDs within what is available, and we can work to implement these standards within what is available…". P3 P3L87.
		"Among the obstacles and challenges is the infrastructure of the ED, which needs to be adapted according to the requirements of the standards, in addition to some shortages in human resources. For example, there is a small laboratory that only works in the morning shift, and the radiology department is in another area of the hospital and needs nursing

		or staff facilities, which is considered a challenge. There is also the lack of a suitable infrastructure close to the staff for hand hygiene". P5 P2L66.
		"Staff shortages, inadequate infrastructure—including the triage department's location, space, and design… are major challenges…". P7 P2L76
		"The staff in our ED possess basic qualifications but require further training in quality improvement, infection control, clinical practices, and EDQS tailored to the Palestinian context…". P10 P3L90.
		"…We also require specialized doctors in Emergency Medicine, as well as nurses and paramedical professionals like lab and radiology technicians". P8 P3L120.
	3.3. Commitment and resistance to change	*"My constant fear is the availability of real administrative support or shortage of personnel and other resources, but these fears can be overcome, for example, in shortage of resources, we sometimes resort to seeking external support…". P6 P4L166.*
		"My concern is about the commitment and support of leadership and management". P8 P4L147.
		"The cadres realize the importance of these standards, but there is a kind of resistance to any change. Another reason is the commitment of the senior management. Despite some resource and logistic challenges, their implementation is possible and has not been a reason to make them unfeasible for implementation". P4 P2L79.

	3.4. Political Situation	*"...Furthermore, the ongoing conflict in Palestine and the increasing injuries from occupation practices place additional pressure on EDs. P7 P2L78.*
	3.5. Workload	*"Our staff faces a heavy workload, which may be the primary concern regarding the additional implementation demands of these standards". P5 P3L123.*
4. Recommendations	4.1. Capacity building and resource allocation	*"The most important recommendation for me is to conduct the orientation and training of these standards for the cadres, ...". P7 P4L165.*
	4.2. Gradual implementation	*"..., I suggest that the experiment should be at the level of an emergency department or two and gradually expand." P6 P5L215.*
		"..., pilot the standards in one or two hospitals before they are widely disseminated or mandatory at the national level,...,". P6 P4L146.
	4.3. Continuous Quality Improvement	*"..., consistently monitoring implementation are crucial for compliance...,". P2 P2L65*
